# Using clustering algorithms to examine the association between working memory training trajectories and therapeutic outcomes among psychiatric and healthy populations

**DOI:** 10.1007/s00426-022-01728-1

**Published:** 2022-09-17

**Authors:** Or David Agassi, Uri Hertz, Reut Shani, Nazanin Derakshan, Avigail Wiener, Hadas Okon-Singer

**Affiliations:** 1 Department of Psychology, School of Psychological Sciences, University of Haifa, Haifa, Israel; 2 The Integrated Brain and Behavior Research Center (IBBR), University of Haifa, Haifa, Israel; 3 Department of Cognitive Sciences, School of Psychological Sciences, University of Haifa, Haifa, Israel; 4 Department of Psychological Sciences, Birkbeck University of London, London, UK

## Abstract

Working memory (WM) training has gained interest due to its potential to enhance cognitive functioning and reduce symptoms of mental disorders. Nevertheless, inconsistent results suggest that individual differences may have an impact on training efficacy. This study examined whether individual differences in training performance can predict therapeutic outcomes of WM training, measured as changes in anxiety and depression symptoms in sub-clinical and healthy populations. The study also investigated the association between cognitive abilities at baseline and different training improvement trajectories. Ninety-six participants (50 females, mean age = 27.67, SD = 8.84) were trained using the same WM training task (duration ranged between 7 to 15 sessions). An algorithm was then used to cluster them based on their learning trajectories. We found three main WM training trajectories, which in turn were related to changes in anxiety symptoms following the training. Additionally, executive function abilities at baseline predicted training trajectories. These findings highlight the potential for using clustering algorithms to reveal the benefits of cognitive training to alleviate maladaptive psychological symptoms.

**Supplementary Information:**

The online version contains supplementary material available at 10.1007/s00426-022-01728-1.

## Introduction

Cognitive training programs have yielded improvements in mental health symptoms. In two recent meta-analyses, computerized cognitive training was found to reduce symptoms among people with depression while also improving their overall cognition (Launder et al., 2021; Woolf et al., 2021). In other meta-analyses, participants with mild cognitive impairment or dementia showed improved cognitive abilities and alleviation of anxiety symptoms (García-Casal et al., 2017; Li et al., 2011). While these promising results have been the basis for many cognitive training studies, the findings have been questioned for their inconsistency across studies (for discussion, see Shani et al., 2019; Okon-Singer, 2018). Diverging outcomes of the training are common, even in studies that have used an identical training program and similar sample characteristics. These diverging outcomes have motivated the suggestion to examine the inherent potential of training dynamics in understanding and explaining the differential outcomes in cognitive training (Könen & Karbach, 2015). The aim of the current investigation was to identify how specific training trajectories are related to training efficacy. To this end, we chose a training program that targets working memory (WM), a system that has limited capacity in storing and processing relevant information (Baddeley & Hitch, 1974) and that influences and is influenced by vulnerability to psychological symptoms, including anxiety and depression (Moran, 2016; Semkovska, 2019; see Derakshan, 2020, for a review).

According to cognitive theories, deficient attentional control plays a key role in maintaining the vicious cycles of depression and anxiety (Berggren & Derakshan, 2013; Eysenck et al., 2007; Joormann & D’Avanzato, 2010; Koster et al., 2017). Attentional control is a dominant feature of WM and is defined as the ability to select only relevant information while ignoring irrelevant material (Duncan & Humphreys, 1989; Unsworth et al., 2012). Studies have suggested that poor ability to filter irrelevant information from WM may lead to psychological symptoms such as worry, anxiety and depression (Derakshan, 2020; Stout et al., 2015; Daches & Mor, 2014; Owens et al., 2013). The Attentional Control Theory (ACT, Eysenck et al., 2007) predicts a compensatory relation between emotion and cognition. Enhanced bottom-up processing undermines top-down regulation, which in turn leads to increased emotional processing. Conversely, when cognitive resources are required to identify a threat, emotional resources dwindle (for a review, see Berggren & Derakshan, 2013).

In recent years, WM training has been shown to play a role in alleviating symptoms of anxiety and depression (Sari et al., 2016; Beloe & Derakshan, 2019; see Derakshan, 2020, for a review). Such training studies usually include one task that serves as the training task and other tasks that are used to examine transfer to other cognitive domains (hereinafter: transfer tasks). Training-related changes as a function of WM training have been found in structurally similar tasks (near transfer). Such changes are also generalized to other untrained, dissimilar cognitive abilities (far transfer), which in turn may reduce anxiety and depression-related symptomatology (Owens et al., 2013; Sari et al., 2016). Nevertheless, inconsistent findings have generated controversy over the efficacy of WM training (for reviews, see Melby-Lervåg et al., 2016; Schwaighofer et al., 2015; Melby-Lervåg & Hulme, 2016; Wanmaker, Geraert & Franken, 2015). These diverging results can be attributed to different designs and methodological preferences (Karbach & Verhaeghen, 2014). Yet even participants who went through the same training task with similar training features demonstrated a high degree of variance in training outcomes (Könen & Karbach, 2015). It is possible that individual differences in training performance may help in understanding the mixed results regarding training effectiveness with respect to psychological symptoms (Könen & Karbach, 2015; Von Bastian & Oberauer, 2014). For example, Hotton et al. (2017) suggested that the absence of training-related improvements may explain the ineffectiveness of symptom reduction among high worriers. More recently, Ciobotaru et al. (2021) showed that training-related effects on improvements in emotional vulnerability were moderated by baseline levels of psychopathology.

Studies that have examined the effect of training progression focused on the training task itself or on other near-transfer cognitive tasks to evaluate training gains (Bürki et al., 2014; Guye et al., 2017). Nevertheless, very few studies have investigated the potential association between training trajectories and improvement in psychological symptoms. Moreover, training studies have adopted different analytical approaches in analyzing training efficacy (e.g., Bürki et al., 2014; Guye et al., 2017), possibly due to different training factors such as training tasks, number of sessions and duration of each session. These differences in analytical approach also highlight the importance of applying a more flexible and adaptive method in analyzing training trajectories.

Individual differences in cognitive abilities at baseline constitute another primary factor that may explain differences across cognitive training performance outcomes. A recent meta-analysis of 16 studies using adaptive WM training contends that near and far transfer cognitive abilities at baseline are consistent negative predictors of WM training outcomes (Ophey et al., 2020). In contrast, findings about training task baseline scores as predictors for training outcomes are more heterogenous (Ophey et al., 2020; Roheger et al., 2021), such that some studies report greater gains for low baseline cognitive ability (e.g., Borella et al., 2017; Zinke et al., 2014) while others report opposite effects (e.g., Brehmer et al., 2011; Heinzel et al., 2014).

In summary, despite the evidence that WM training may alleviate symptoms of anxiety and depression, findings are mixed. Most of the research so far has focused on the impact of training on behavioral outcomes and not on individual differences in learning patterns during training and on how these differences may be related to training efficacy and effects on well-being. To fill this gap, the current investigation sought to improve the understanding of WM training efficacy by examining inter-individual differences in learning patterns during training.

We collected data from six studies conducted in our laboratory and by collaborators, all using the same dual n-back training task. The dual n-back is an online computerized task that has been widely used in recent years to improve WM and other aspects of cognitive abilities (see Au et al. 2015, for a review). The studies we included used different pre-selection criteria and training durations, enabling us to examine the association between potential factors and training outcomes. Each study included at least one emotional questionnaire and transfer task in the pre- and post-training assessments. To define different learning curve trajectories, we used k-means, an innovative algorithm in this field, to cluster participants with homogenous learning characteristics. Finally, we examined the differences in psychological symptoms between these learning clusters as well as the potential predictors of the learning trajectories. Implementing a large-scale, diverse dataset enabled us to optimize the definition of varied training trajectories.

Given the sparsity of research and controversial disagreements in the field of inter-individual differences in cognitive training, we adopted an exploratory approach in our analyses. Based on diverging results in training outcomes, even for the same training regimes, we expected to find several trajectories for the dual n-back training that are associated with different psychological symptoms. Moreover, we predicted that learning trajectories are related to differences in cognitive abilities at baseline.

## Methods

### Participants

Six datasets were collected, comprising a total of 96 participants. The data included five datasets from the lab of Nazanin Derakshan (Course-Choi et al., 2017; Ducrocq et al., 2017; Hotton et al., 2017; Owens et al., 2013; Sari et al., 2016) and one from a study conducted by Wiener and Okon-Singer (unpublished results). The dropout rates ranged between 8 and 20%. All these studies included the following information: demographic details, pre-post emotional questionnaires, and pre-post cognitive tasks (Table 1). Three of the studies also included a 4-week follow-up measurement that entailed questionnaires and tasks. The data encompass participants who exhibit a large variety of mental disorders, among them anxiety, sub-clinical depression and worry, as well as healthy participants. Training sessions ranged from 7 to 15 days. Table 1 depicts the characteristics of all datasets.

**Table 1:** Characteristics of All Datasets

Study by year		*N* (female)	Pre-selection condition	Question-naires (pre, post, FU*)	Cognitive tasks (pre, post, FU*)	Age (std. dev.)	Training duration
Owens, Koster & Derakshan (2013)		11 (7)	BDI-II score ≥ 20	BDI	CDT	25.27 (5.33)	8 sessions
Sari, Koster, Pourtois & Derakshan (2016)		13 (8)	STAI trait Anxiety ≥ 50 ACS ≤ 60	STAI PSWQ	Flanker task (interference scores)	22.81 (4.47)	15 sessions
Hotton, Derakshan & Fox (2017)		20 (16)	PSWQ ≥ 56	PSWQ* STAI*	CDT* Flanker task*	29.2 (11.75)	15 sessions
Ducrocq, Wilson, Smith & Derakshan (2017)		13 (3)	Recreational tennis players	SAQ	CDT	34.77 (13.29)	10 sessions
Wiener & Okon-Singer	Unpublished results	24 (6)	Prehypertensive	STAI* BDI* PSWQ* RRS*	CDT* Flanker task*	25.92 (3.53)	14 sessions
Course-Choi, Saville & Derakshan (2017)		15 (10)	PSWQ ≥ 45	STAI* PSWQ* RRS*	CDT	27.93 (7.29)	7 sessions
Total		96 (50)				27.67 (8.84)	

All the WM study datasets used dual N-back training and included psychological symptoms questionnaires, demographics, and cognitive tasks

*BDI* beck depression inventory, *CDT* change detection task, *FU* follow up, *PSWQ* penn state worry questionnaire, *RRS* ruminative response scale, *SAQ* sports anxiety scale, *STAI* state-trait anxiety inventory, *WM* working memory

*Includes a 4-week follow-up measurement

### Instruments and measurements

#### Cognitive tasks

Participants completed a daily practice session that entailed a computerized dual n-back task (Jaeggi et al., 2008) installed on their personal PCs. In this training task, the participant is simultaneously confronted with both an auditory and a visual stimulus and must decide whether both, one or none of the stimuli match the stimuli that were presented *n *items back, where *n* is changed adaptively from 1 to 4 according to the participant’s performance. In addition to the training task, each participant’s cognitive abilities were tested before and after the training. To examine the near-transfer effect, we implemented the change detection task (CDT; Owens et al., 2013). This task, adapted from Vogel et al. (2005), measures visuospatial working memory. To measure the far-transfer effect, we assessed inhibition using a modified version (Berggren & Derakshan, 2013) of the original flanker task (Eriksen & Eriksen, 1974). More details about the training and transfer tasks are provided in the supplementary material.

#### Psychological symptoms indices

All participants completed at least one questionnaire before and after the training to assess training-related improvement in emotional symptoms. The included studies employed different questionnaires to measure symptoms of anxiety and depression. In order to collapse these measures across studies, we created index scores based on several questionnaires. The rationale for creating main indices is the desire to use as much of the available data as possible. These indices allow for the use of large-scale and diverse data to examine relations between training performance and changes in psychological symptoms. The three main indices were created while taking into account statistical and theoretical issues (for a similar approach, see Rohr et al., 2015). The *anxiety index* consisted of the State-Trait Anxiety Inventory (STAI; Spielberger et al., 1970), the Penn State Worry Questionnaire (PSWQ; Meyer et al., 1990), and the Sports Anxiety Scale-2 (SAS-2; Smith et al., 2006) questionnaires, while the *depression index* included the Beck Depression Inventory-II (BDI-II; Beck et al., 1996) and the Ruminative Responses Scale (RRS; Nolen-Hoeksema & Morrow, 1991) questionnaires.

The final index—the *general psychological symptoms* index—was computed as the average of the scores on both the anxiety and the depression indices. For details about the questionnaires, see the supplementary material.

### Data analysis

#### Defining learning trajectories

The k-means clustering algorithm was used to facilitate data-driven identification of training trajectories classes. For this purpose, we used the “kml” package for R (version 3.5.3; R Core Team, 2018), which is designed for working with longitudinal data (version 2.4.1; Genolini et al., 2015). The k-means algorithm belongs to the EM class (expectation–maximization; Celeux & Govaert, 1992). Depending on the initial configuration chosen, after k cluster centers have been set, the algorithm clusters all the training trajectories to k-number of clusters based on the minimal distance between the trajectory and each cluster center. In the expectation phase, the central position of each cluster, also called the seed, is computed. During the maximization phase, all training trajectories are reassigned to their nearest cluster based on the distance to its seed. Regarding studies with shorter training durations, the algorithm calculates the main learning clusters based on all training trajectories and available data, even from shorter trajectories. These two phases are repeated until none of the observations in each cluster change on the next partition. It is important to note that the main training trajectories are not limited to any specific model type such as linear, non-linear or quadratic models. The trajectories are computed as the average score of all trajectories in the cluster. This fluid algorithm enables us to define different types of trajectories that provide better discrimination between trajectories clusters and show smaller differences within each cluster.

We ran the algorithm with its default settings: The initial configuration to k-cluster centers was k means +  + (Arthur & Vassilvitskii, 2007) and the EM phases were run 20 times for each number of clusters. After the algorithm was run, the first step was to choose the fittest number of clusters for this specific study, ranging from 2 to 6. We strove to achieve the highest number of clusters possible to capture as much of the variance in the data as possible. Clusters with exceptionally unequal groups (six clusters) were eliminated to avoid groups that were too small. Finally, several quality indices were examined for each number of clusters to decide the right number of clusters.

After defining the number of the main trajectories, we selected the iteration with the highest score (out of 20) according to the Calinski and Harabasz criterion (Calinski & Harabasz, 1974): ${\text{C}}\left( {\text{k}} \right)\, = \,\frac{{{\text{Trace}} \left( B \right)}}{{{\text{Trace }}\left( W \right)}} \cdot \frac{n - k}{{k - 1}}$ where *B* is the between-cluster covariance matrix and *W* is the within-cluster covariance matrix. Higher values of Trace (*B*) and lower values of Trace (*W*) are a strong indication of correct clustering.

To better understand each trajectory and its unique nature, the study examined the differences between the training score trajectories on the first day (i.e., baseline), the score on each training day and the level of daily improvement on the trained task (i.e., slope). Training task improvement was based on each participant’s score on each training cluster on the last training session day minus the score on the first day. These effects were analyzed using ANOVA and by a post-hoc Tukey test in case of significance. Due to the exploratory nature of these analyses, we did not correct for multiple comparisons.

#### Cross-validation of the main trajectories

To validate our results, we ran a cross-validation test on the entire dataset (Farrell & Lewandowsky, 2018). Data were split based on odd or even participant numbers to create two mixed, equally numbered sub-groups of 48 participants each. We used each group as a training group and applied the *k*-means algorithm for clustering into three main training patterns. The participants in each test group—the odd-numbered group and the even-numbered group—were clustered according to their training trajectories.

#### Learning performance and psychological symptoms

After the main trajectories clusters were defined, we examined differences in psychological index improvement between all clusters. To this end, we used ANOVA to examine the relationship between training trajectory classes and improvement in the *anxiety index*, *depression index,* and *general psychological symptoms index* (pre-post). Improvement was defined as the separate change scores on each of these three indices. When the original study included follow-up measurements, we ran the same tests on the follow-up measurements to examine the long-term effects of the training on psychological symptoms. Tests and diagrams were conducted using the “ggpubr” package (Kassambra, 2018) in R. Significant results were analyzed using a post-hoc Tukey test, taking into account Bonferroni correction. In case of a between-group difference on one of the measures, an additional *T* test was conducted to examine whether this measure improved significantly in each of the groups.

#### Predicting clustering by demographic details, cognitive abilities and psychological symptoms

An ANOVA was run to examine the association between training clusters and pre-training cognitive abilities, namely WM (assessed by the CDT task) and inhibition (assessed by the flanker task). Similarly, we tested whether training trajectories can be predicted by any of the psychological symptom indices, namely, anxiety, depression, and general psychological symptoms indices. A post-hoc Tukey test was used to analyze significant results. Finally, to check whether demographics are a factor in the prediction of learning clusters, the same analyses were conducted on gender and age.

#### Mediation analysis

Based on our results, we fitted a structural equation model (SEM) to estimate the association between training performance and inter-individual differences in cognitive abilities and well-being. SEM analyses were conducted using the "lavaan" package (version 0.6-4; Rosseel, 2012) in R. To investigate all the relationships, we built a mediation model using SEM. In our model, maximum likelihood (ML) was used to overcome missing data. We compared our model to a baseline model that included the mean and variance of all observed variables plus the covariances of all observed exogenous variables.

Please note that the methodological process in this study was exploratory. Consequently, no pre-registration protocol was applied for this study.

## Results

### Defining learning trajectories

**Fig. 1:**
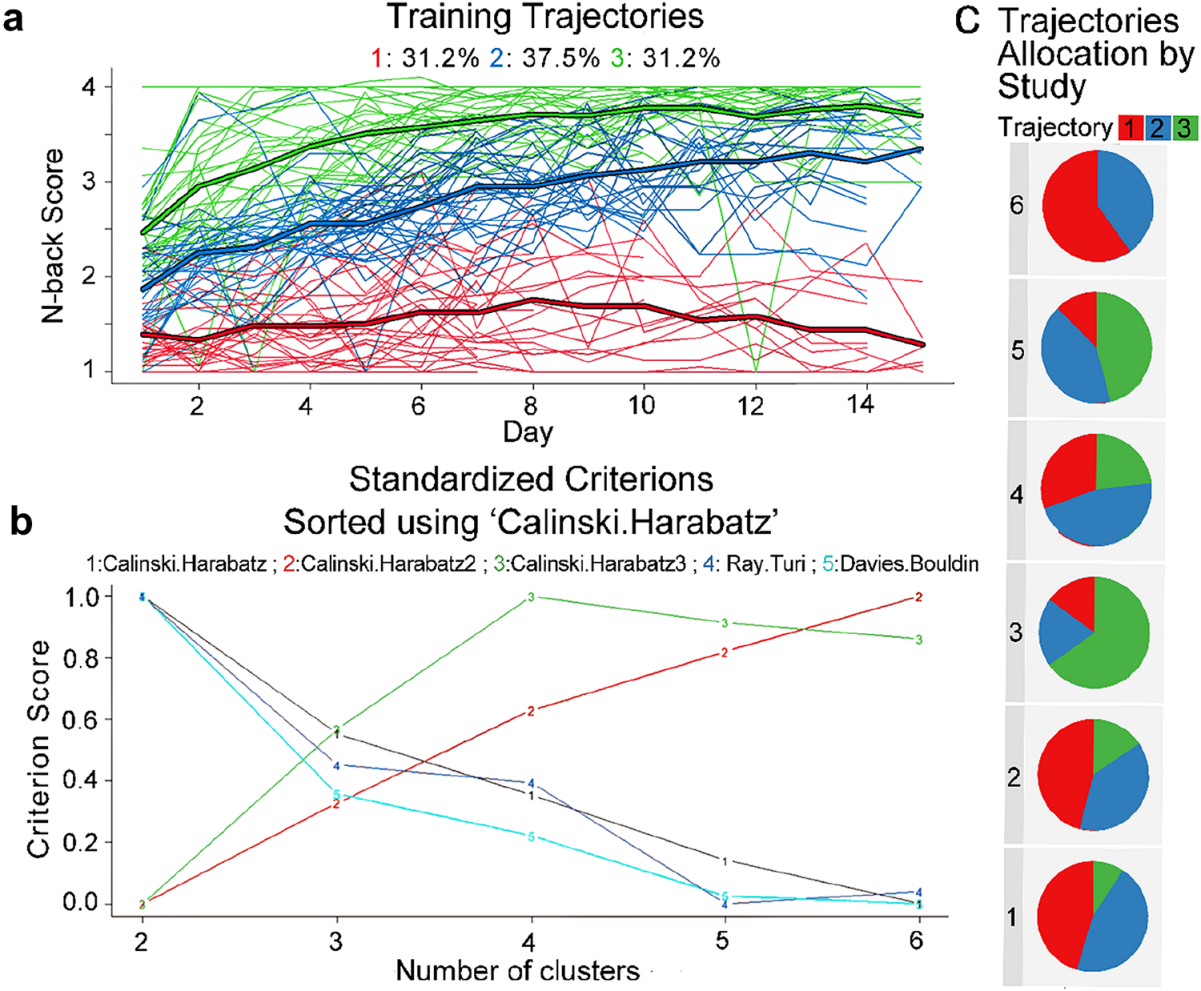
Defining Training Trajectories. **a** All trajectories were grouped into three main clusters (trajectories) as suggested by the algorithm. Cluster 1 (red) did not show any improvement, cluster 2 (blue) demonstrated consistent improvement, and cluster 3 (green) demonstrated non-linear improvement. **b** Quality criteria for score/time trajectories. Each line represents a criterion score for each cluster number (possible range 2–6). Standardized criteria all agreed on an intermediate score for three clusters. **c** Participants in each of the three clusters (red, blue, and green) were present in (almost) all datasets

Three trajectories were defined (Fig. 1A) based on the following rationale: First, the three-cluster allocation achieved an intermediate score on each criterion of the algorithm, better than any of the other allocations. Second, the three-cluster allocation yielded groups almost of equal size, suggesting that this allocation was not caused by a very small group. Criteria scores for the three clusters were: “(1) Calinski Harabatz-1: 137.12; (2) Calinski Harabatz-2: 3.01; (3) Calinski Harabatz-3: 193.92; (4) Ray Turi: − 0.03; (5) Davies Bouldin: − 1.20. For standardized criteria, see Fig. 1B. The three clusters were characterized as follows: The first cluster (cluster 1) included 30 participants (31.2%) of the 96. As can be seen in Fig. 1A (red line), most of these participants did not show any improvement during the training sessions. This main trajectory begins with a mean score of *n* = 1.39, followed by a slight improvement until session 8 (*n* = 1.76). This improvement is not maintained, as the trajectory finishes with a mean score of *n* = 1.28. The second trajectory cluster (cluster 2; blue line) included 36 participants (37.5%). This trajectory begins with a higher score than the first cluster, with a mean score of *n* = 1.86. In addition, throughout all the training days, this trajectory cluster was significantly higher than the trajectory in cluster 1. The trajectory improved consistently over the sessions until reaching an n-score of 3.34. The third cluster (cluster 3) also included 30 participants (31.2%) who showed the highest mean score in each session of the training. This cluster starts with the highest training score, with a mean of *n* = 2.46. The progression of this trajectory is slightly curved and ends with a score of *n* = 3.70, which was statistically different from the score on the last day in cluster 1 but not in cluster 2. For a comprehensive comparison between training trajectories please see the supplementary material.

To verify that this clustering did not result from the specific studies included in our sample, we checked how the clustering was applied to each of our base studies. The studies included participants from all three clusters, except for one study that had participants from only two training clusters (Fig. 1C). Please note that the inclusion criterion for this study was similar to that of another study (i.e., participants with high scores on a worry questionnaire).

The amount of improvement on the training task differed between the training trajectories $\left[ {F_{{\left( {2,93} \right)}} = 36.1, \,p < 0.0001, \,\eta^{2} = 0.437} \right]$. In a post-hoc Tukey-HSD, the improvements in training cluster 3 $\left[ {M = 1.32, \,SD = 0.577} \right]$ and cluster 2 $\left[ {M = 1.28, \,SD = 0.692} \right]$ were higher than those in training cluster 1 $\left[ {M = 0.171, \,SD = 0.508} \right]$. The training trajectories also differed from each other on the first-day score of the training task $\left[ {F_{{\left( {3,93} \right)}} = 43.73, \,p < 0.0001,\, \eta^{2} = 0.485} \right]$, such that participants in training cluster 3 had a higher average first-day score $\left[ {M = 2.46, \,SD = 0.506} \right]$ than participants in cluster 2 $\left[ {M = 1.86,\, SD = 0.468} \right]$. Moreover, participants in training cluster 2 scored higher on the training task than participants in cluster 1 $\left[ {M = 1.39, \,SD = 0.338} \right]$.

### Cross-validation of the main trajectories

In a cross-validation test of the main trajectories, both training groups (i.e., datasets from the odd and the even participants) exhibited trajectory lines similar to those found based on all the data. Specifically, 91 out of the 96 participants were clustered to the same learning trajectory to which they had been clustered based on the full data. Percentages of participant allocation to each cluster were also similar, with only small variations. In the odd training group, allocation percentages to clusters 1–3 were 29.2, 33.3, and 37.5%, respectively, while in the even training group allocation percentages to clusters 1–3 were 27.1, 39.6, and 33.3%, respectively.

### Learning performance and psychological symptoms

To examine the effect of training performance on psychological symptoms, we used repeated-measures ANOVAs. In each ANOVA, the independent variable was the learning clusters, and the dependent variable was the change in each psychological symptom index (pre-post).

**Fig. 2:**
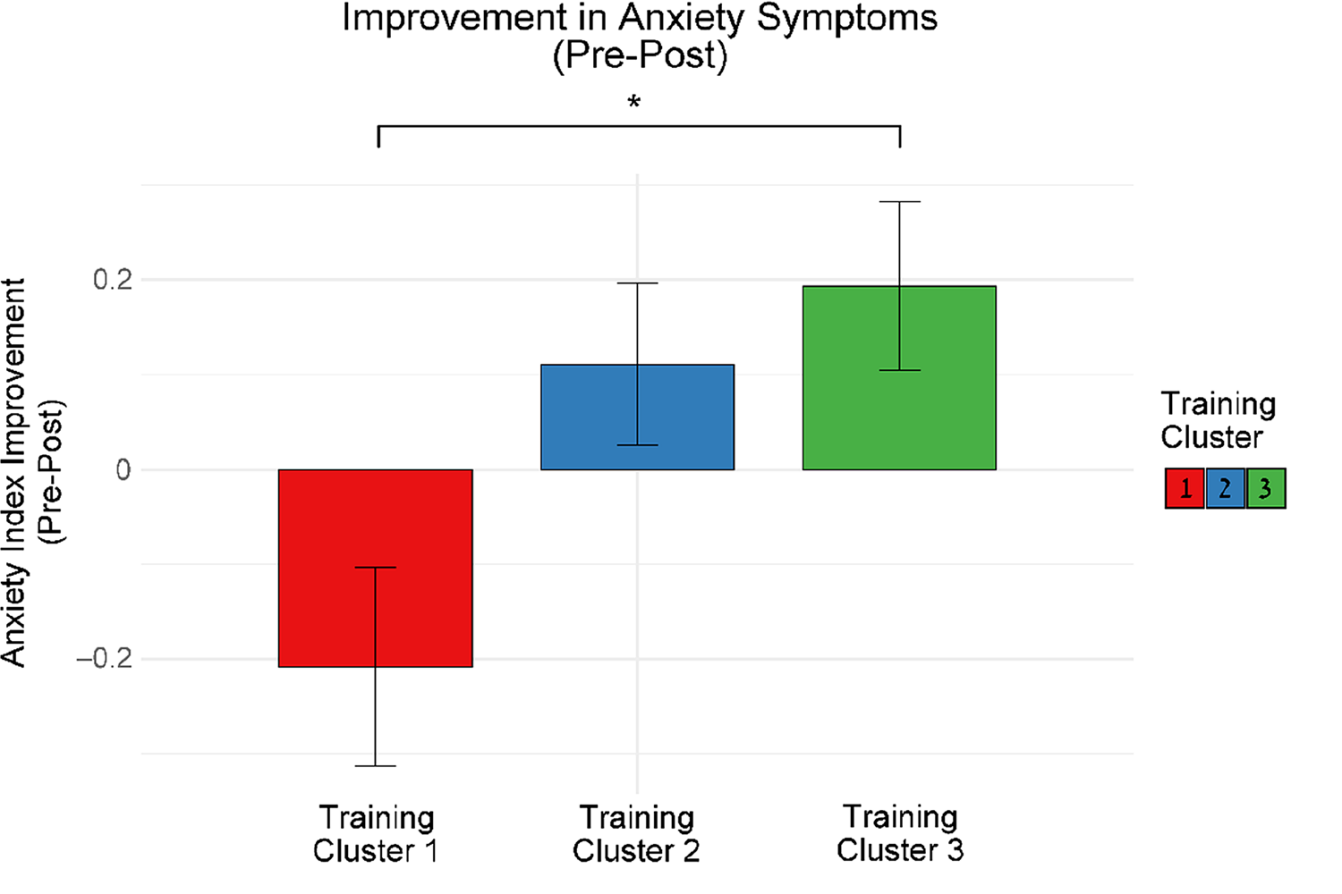
Improvement in anxiety index pre-post training (T1-T2). Participants in cluster 3 showed more improvement than participants in cluster 1. Error bars show standard errors

Improvement in anxiety symptoms from pre- to post-training differed between learning clusters (Fig. 2) $\left[ {F_{{\left( {2,81} \right)}} = 4.854, \,p < 0.05, \,\eta^{2} = 0.107} \right]$. Yet no difference between clusters emerged for change in depression symptoms $\left[ {F_{{\left( {2,47} \right)}} = 0.103, \,p = 0.902, \,\eta^{2} = 0.004} \right]$ or for general psychological symptoms $\left[ {F_{{\left( {2,92} \right)}} = 1.433, \,p = 0.244, \,\eta^{2} = 0.03} \right]$. Furthermore, Tukey-HSD testing was used for post-hoc comparisons, taking into consideration alpha inflation by using Bonferroni correction. Our analysis indicated that the improvement in anxiety symptoms among participants in cluster 3 $\left[ {M = 0.193, \,SD = 0.478} \right]$ was greater than among those in cluster 1 $\left[ {M = - 0.208, \,SD = 0.513} \right]$.

**Fig. 3:**
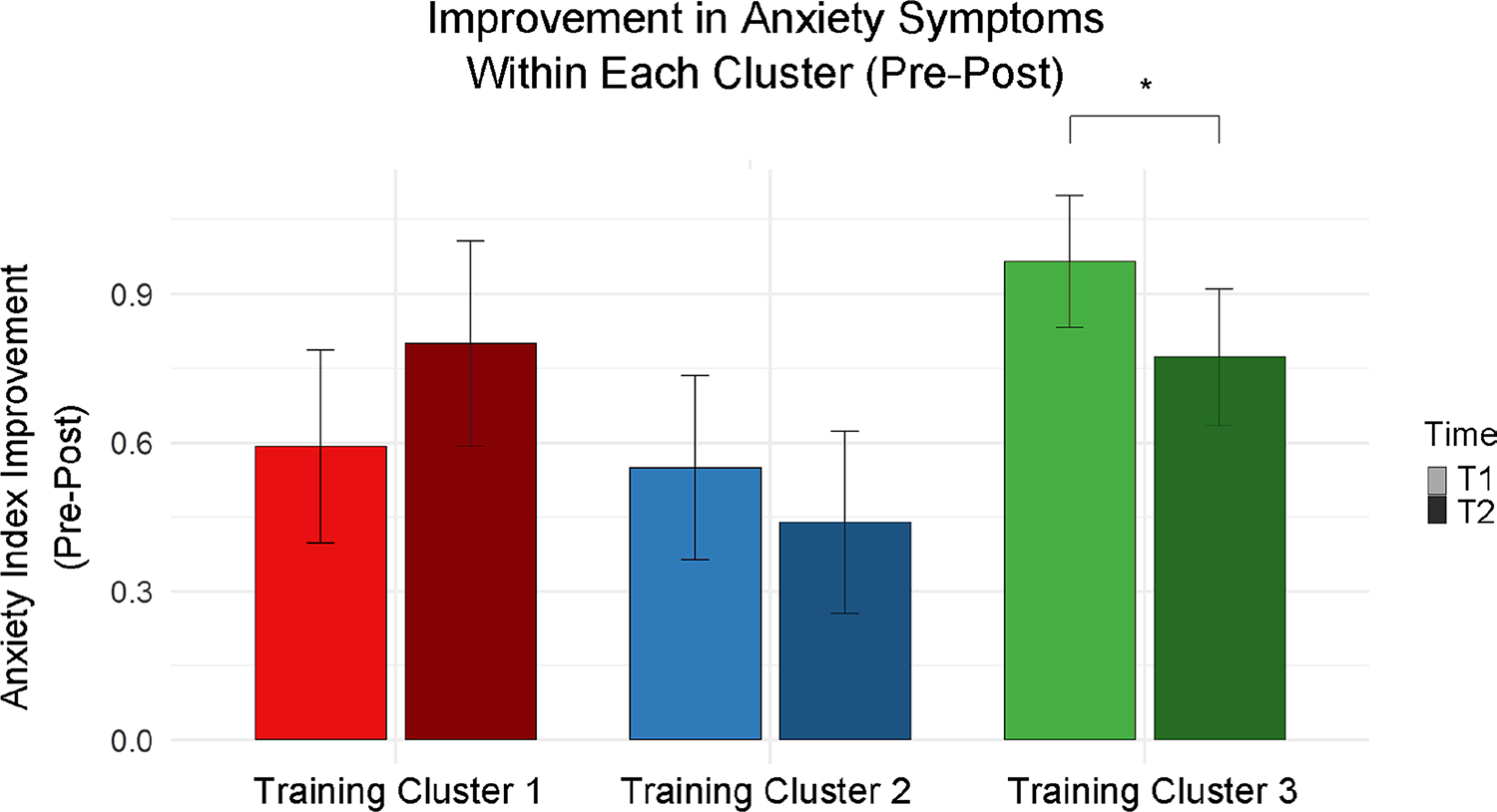
Intra-group improvement in anxiety symptoms pre-post training (T1-T2). Only participants in cluster 3 demonstrated a reduction in anxiety symptoms. Error bars show standard errors

T-tests that examined the change in anxiety symptoms in each cluster showed that anxiety symptoms decreased only in cluster 3 $\left[ {T_{{\left( {28} \right)}} = 2.179, \,p < 0.05, \,Cohen^{\prime}s d = 0.405} \right]$ (Fig. 3).

We conducted ANOVAs on follow-up measurements to identify differences between learning clusters on long-term improvements in psychological symptoms. The analyses indicated a difference in anxiety symptom improvements even over the long term $\left[ {F_{{\left( {2,54} \right)}} = 6.062, \,p < 0.01, \,\eta^{2} = 0.183} \right]$. In contrast, long-term improvements in depression $\left[ {F_{{\left( {2,45} \right)}} = 0.575, \,p = 0.567, \,\eta^{2} = 0.025} \right]$ and in general psychological symptoms $\left[ {F_{{\left( {2,65} \right)}} = 1.398,\, p = 0.254, \,\eta^{2} = 0.041} \right]$ were not related to cluster allocation. In a post-hoc analysis for anxiety symptoms, the Tukey-HSD analysis demonstrated a significant difference between learning cluster 3 $\left[ {M = 0.380,\, SD = 0.431} \right]$ and cluster 1 $\left[ {M = - \,0.270, \,SD = 0.825} \right]$.

### Predicting clustering by demographic details, cognitive abilities and psychological symptoms

An examination to determine whether demographic details, cognitive abilities or pre-training psychological symptoms predicted clusters found that clusters were not related to gender, age or psychological symptoms (Table 2). Nevertheless, the pre-training score on the CDT task differed between clusters $\left[ {{\varvec{F}}_{{\left( {{\mathbf{2,79}}} \right)}} = {\mathbf{5}}{\mathbf{.164}},\,{ }{\varvec{p}} < {\mathbf{0}}{\mathbf{.01}},\,{ }{\varvec{\eta}}^{{\mathbf{2}}} = {\mathbf{0}}{\mathbf{.116}}} \right]$. Post-hoc multiple comparisons revealed a higher score for participants in cluster 3 $\left[ {{\varvec{M}} = {\mathbf{1}}{\mathbf{.73}},{ }{\mathbf{SD}} = {\mathbf{0}}{\mathbf{.447}}} \right]$ than for those in cluster 1 $\left[ {{\varvec{M}} = {\mathbf{1}}{\mathbf{.03}}\,,{ }{\mathbf{SD}} = {\mathbf{1}}{\mathbf{.09}}} \right]$. In addition, the pre-training flanker scores differed between clusters $\left[ {{\varvec{F}}_{{\left( {{\mathbf{2,50}}} \right)}} = {\mathbf{9}}{\mathbf{.274}},\,{ }{\varvec{p}} < {\mathbf{0}}{\mathbf{.001}},\,{ }{\varvec{\eta}}^{{\mathbf{2}}} = {\mathbf{0}}{\mathbf{.271}}} \right]$, such that participants in cluster 3 had a better flanker score $\left[ {{\varvec{M}} = {\mathbf{41}}{\mathbf{.2}},\,{ }{\mathbf{SD}} = {\mathbf{28}}{\mathbf{.1}}} \right]$ than participants in cluster 2 $\left[ {{\varvec{M}} = {\mathbf{78}}{\mathbf{.4}},\,{ }{\mathbf{SD}} = {\mathbf{35}}{\mathbf{.7}}} \right]$ and in cluster 1 $\left[ {{\varvec{M}} = {\mathbf{79}},\,{ }{\mathbf{SD}} = {\mathbf{32}}{\mathbf{.7}}} \right]$.

**Table 2:** Demographic Details, Cognitive Abilities and Psychological Pre-training Symptoms: Comparison Between Clusters

		ANOVA	Post-hoc
Age		${\varvec{F}}_{{\left( {\user2{2,93}} \right)}} = {\mathbf{2}}{\mathbf{.363}},\user2{ P} = {\varvec{n}}.{\varvec{s}}.$	***n.s***
Gender		${\varvec{F}}_{{\left( {{\mathbf{2,93}}} \right)}} = {\mathbf{0}}{\mathbf{.795}},\user2{ P} = {\varvec{n}}.{\varvec{s}}.$	***n.s***
Cognitive abilities	CDT	${\varvec{F}}_{{\left( {{\mathbf{2,79}}} \right)}} = {\mathbf{5}}{\mathbf{.164}},\user2{ P} < {\mathbf{0}}{\mathbf{.01}},\user2{ n}^{{\mathbf{2}}} = {\mathbf{0.116}}$	***3* > *1, p* < *0.05***
	Flanker	${\varvec{F}}_{{\left( {{\mathbf{2,50}}} \right)}} = {\mathbf{9}}{\mathbf{.274}},\user2{ P} < {\mathbf{0}}{\mathbf{.001}},\user2{ n}^{{\mathbf{2}}} = {\mathbf{0}}{\mathbf{.271}}$	***3* > *1, p* < *0.01*** ***3* > *2, p* < *0.05***
Psychological symptoms	Anxiety	${\varvec{F}}_{{\left( {{\mathbf{2,81}}} \right)}} = {\mathbf{1}}{\mathbf{.815}},\user2{ P} = {\varvec{n}}.{\varvec{s}}.$	***n.s***
	Depression	${\varvec{F}}_{{\left( {{\mathbf{2,47}}} \right)}} = {\mathbf{0}}{\mathbf{.795}},\user2{ P} = {\varvec{n}}.{\varvec{s}}.$	***n.s***
	General psychological symptoms	${\varvec{F}}_{{\left( {{\mathbf{2,92}}} \right)}} = {\mathbf{0}}{\mathbf{.182}},\user2{ P} = {\varvec{n}}.{\varvec{s}}.$	***n.s***

Table 2 shows a comparison of possible predictors for training trajectories. Only cognitive abilities were related to training clusters, as participants in cluster 3 scored higher on near and far transfer tasks

### Mediation analysis

**Fig. 4:**
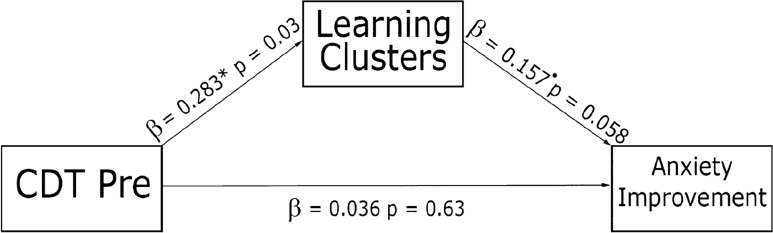
The relations among cognitive abilities, learning clusters and improvement in anxiety symptoms. CDT baseline score exhibited a positive relation to learning performance. Moreover, learning performance exhibited a marginally significant positive relation to improvement in anxiety symptoms

Based on the findings of an effect of learning clusters on anxiety symptoms and of a relation between pre-training cognitive abilities and training trajectories, a mediation model (Fig. 4) was used to examine whether training improvement mediated between cognitive abilities and anxiety improvement. Due to an insufficient number of participants who performed a flanker task, we used only the CDT task scores (82 observations).

The model was found to fit better than the baseline model $[\chi_{\left( 3 \right)}^{2} = 13.289, \,p < 0.01]$. Pre-training CDT scores predicted training clusters $[\beta = 0.283,\, SD = 0.096,\, Z = 2.940,\, p < 0.01]$ and a marginally significant relation emerged between training performance (cluster) and anxiety improvement $\left[ {\beta = 0.157,\, SD = 0.083,\, Z = 1.893,\, p = 0.058} \right]$. CDT scores pre-training did not directly predict anxiety improvement $\left[ {\beta = 0.036,\, SD = 0.075,\, Z = 0.482,\, p = 0.630} \right]$.

## Discussion

The current study aimed to explain WM training efficacy by means of learning trajectories. We examined training performance patterns and their relation to improvement in well-being. The main finding of this study was that patterns of training performance were highly related to anxiety improvement following the training. Specifically, a sharp learning curve at the early stages of WM training combined with a higher baseline training score explained improvement in anxiety symptoms. Consistent with previous research (Guye et al., 2017), we also found that learning trajectory mediated the relation between cognitive abilities at baseline and the impact of training on anxiety symptoms.

Understanding training trajectories can contribute to building tailored training programs based on patient characteristics (Shani et al., 2019). In the last decade, the research domain of personalized treatment based on individual characteristics has grown substantially (Zilcha-Mano, 2019). To build an optimal training trajectory for a sample of patients with similar characteristics, training settings should be continually adapted during the sessions to maximize training effectiveness. Likewise, constant deviations from an optimal training trajectory during the first sessions can indicate that the cognitive task is ineffective for the individual and suggest the need for modification or replacement of the training program.

In this exploratory study, we used a *k*-means algorithm for longitudinal data to define different trajectories in the dual n-back training. To the best of our knowledge, the use of this semi-automatic algorithm is unprecedented in this field and provides a new perspective for examining WM training. Moreover, based on our data, we generated three psychological symptom indices to investigate the relation between training trajectories and improvement in the symptoms of anxiety, depression and general psychological symptoms. We believe that using this algorithm can enhance our understanding of who benefits more from cognitive trainings and can thus enable us to utilize training gains.

Our results show that training trajectories can be clustered to a small number of main training trajectories that differ on baseline training task score, learning curve and degree of improvement. Moreover, these training trajectories were robust in a strict cross-validation test. These results can help us understand the high variance of individual differences during training and how this variance is related to training outcomes (Hotton et al., 2017; Könen & Karbach, 2015). It is important to note that we defined the trajectories based only on the training scores while ignoring the original study from which they were collected. This method is designed to find more general clusters that can be implemented on other cognitive training tasks for different cognitive skills, for example, cognitive bias modification (CBM) training, which has demonstrated questionable efficacy (Cristea et al., 2015).

Our findings show that executive inhibitory abilities at baseline are positively related to training outcomes. Indeed, recent empirical studies indicated a strong relationship between individuals with high cognitive abilities and WM training gains (Guye et al., 2017; Wiemers et al., 2019). Nevertheless, a mediation test model built on our data revealed that learning trajectories may mediate the relation between WM abilities at baseline and post-training improvement in anxiety symptoms. Furthermore, expansion of this analysis demonstrated that training trajectories explain training outcomes above baseline and last training day scores in terms of effect size (see supplementary materials). This finding underscores that learning trajectories may serve as a potential factor in understanding who benefits more from the clinical implications of this type of training.

One interesting finding of this study is that a considerable proportion of individuals do not show training improvements throughout the course of training. Stout, Snackman & Larson, (2013) proposed that anxiety symptoms are related to impaired WM ability due to ineffective filtering of threat-related cues. Therefore, it is likely that poor performance on the WM training task may explain the mixed findings regarding the effectiveness of training on psychological symptoms (e.g., Hotton et al., 2017). More cognitive training studies are needed to explore training trajectories to shed more light on training efficacy.

Our study points to some hypothetical explanations as to why participants in the non-linear training trajectory (Fig. 1, green trajectory) exhibited better training gains. As both the non-linear and the linear (Fig. 1, blue trajectory) trajectories exhibited an identical level of improvement on the n-back task, and no statistical difference was found between these two training trajectories on their last training day score, it is possible that training outcomes are achieved only by a combination of higher pre-training cognitive abilities and a certain level of training gain. Alternatively, a thorough examination of the non-linear improvement trajectory reveals a ceiling effect, as many of the participants who were clustered around this trajectory achieved the maximum score possible on the task. Before each training session, participants in all experiments were told what n-back level they should act upon. This way, participants knew inherently whether they had progressed or deteriorated during the training. Thus, progression during the training was intrinsically rewarding for the participants who improved during the training and especially for those who reached the highest possible level in the task. Consequently, participants in this learning pattern received more rewarding feedback compared to the other trajectories. Participants in the non-linear trajectory may have experienced an increased sense of self-efficacy during the sessions. It has long been established that self-efficacy plays a central role in the self-regulation of mental disorders (Bandura, 1997; Muris, 2002). Self-efficacy is also critical for training motivation, which is known to be an antecedent for training outcomes (Colquitt et al., 2000). Furthermore, we tested the relation between the clusters and the improvements in the flanker task and the CDT scores. These analyses demonstrated a main effect for the training that improved the CDT scores only. In contrast, the improvement in CDT scores was unrelated to the clusters, suggesting that all clusters showed similar improvement in WM. This finding may provide indirect support for the possibility that self-efficacy plays a role in reducing anxiety symptoms (see supplementary materials). Since we did not examine self-efficacy, this suggestion should be considered with caution. Future studies can manipulate self-efficacy directly, for example by using different types of feedback or manipulating the difficulty of the training to examine the effect on anxiety improvement.

Despite the uniqueness of our study, a few limitations should be noted. First, all included studies used an adaptive dual n-back task limited to 4-back, even though most participants are prone to reach level four around session 7 (Jaeggi et al., 2008). Consequently, it is likely that there is an artificial ceiling effect that does not allow participants to reach their maximum potential. It is possible that without any artificial ceiling effect, some changes within the trajectories might occur, especially on the third trajectory. Second, in our mediation model test, training trajectories exhibited a marginally significant relation to improvement in anxiety symptoms. Yet it is reasonable to assume that this relation stems from an insufficient number of observations, as our model used only 82 participants. Another explanation can be derived from the inability of the lavaan package to deal with nominal scales (Rosseel, 2012). Finally, in this study the learning clusters showed no significant difference between depression improvement and general psychological symptom improvement. It is likely that the small number of participants with depression symptoms (only 12 had mild depression according to their BDI-II score) made it difficult to detect such improvement. Hence, caution should be taken with respect to this analysis.

Although this study does not strive to reach a final and conclusive decision regarding training effectiveness, as a proof of concept it lays the groundwork for future studies to investigate the relations between training trajectories and training outcomes. To determine a causal relation, future studies can manipulate training trajectories for each participant by controlling individual training levels to facilitate comparisons with the current results.

To the best of our knowledge, this is the first study to examine the effect of learning trajectories and their association with mental health improvements. Our findings point to a medium to strong relationship between different learning patterns and anxiety symptoms. This proof-of-concept study highlights that learning patterns play a fundamental role in the clinical cognitive training field and provides insight into individual differences in benefiting from cognitive training. Therefore, we believe that learning patterns should be a key factor in developing an adaptive tailored cognitive training program. Future studies can advance our work by examining learning trajectories using other cognitive training paradigms and larger sample sizes to determine their importance in attaining effective training outcomes.

## Supplementary Information

Below is the link to the electronic supplementary material.

Supplementary file1 (DOCX 895 KB)
