# Supplementary material for: Using clustering algorithms to examine the association between working memory training trajectories and therapeutic outcomes among psychiatric and healthy populations
Source: Psychol Res. 2022 Sep 17;87(5):1389–400. doi: 10.1007/s00426-022-01728-1 (PMC10227123; doi:10.1007/s00426-022-01728-1)
Supplement: Supplementary file 1 — Supplementary file1 (DOCX 895 KB) [file 426_2022_1728_MOESM1_ESM.docx]

**Using Clustering Algorithms to Examine the Association between Working Memory Training Trajectories and Therapeutic Outcomes among Psychiatric and Healthy Populations**

Psychological Research

Or David Agassi^a,b^, Uri Hertz^c^, Reut Shani^a,b^, Nazanin Derakshan^d^, Avigail Wiener^a,b^, Hadas Okon-Singer^a,b^
a – Department of Psychology, School of Psychological Sciences, University of Haifa, Haifa, Israel

b – The Integrated Brain and Behavior Research Center (IBBR), University of Haifa, Haifa, Israel

c – Department of Cognitive Sciences, School of Psychological Sciences, University of Haifa, Haifa, Israel

d – Department of Psychological Sciences, Birkbeck University of London, London, UK

Correspondence: Or David Agassi –odagassi@gmail.com

**Supplementary Material**

**Method**

**Instruments and Measurements**

***Cognitive Tasks***

**Training Task:** Dual N-Back Task (Jaeggi et al., 2008). This computerized task simultaneously presents both auditory and visual stimuli. Every three seconds, a green square appears on a screen of 3*3 squares, while at the same time a letter (c, h, k, l, q, r, s, and t) is read aloud. The participant is asked to decide whether one, both or none of the items match the stimuli that had been presented *n* items previously, where *n* is changed adaptively from 1 to 4 depending on the participant’s accuracy. The value of *n* increases when performance is high (95% or above) and decreases when performance worsens (75% or less). This way, the task is tailored to each participant and remains challenging throughout the sessions. The version of the dual n-back task we used was limited to n=4. Each day, participants had to complete 15 blocks, each containing 20+n trials. The score for each day (session) was calculated as the average n scores of all 15 blocks per day.

**Near-Transfer Task:** Change Detection Task (CDT; Owens, Koster & Derakshan, 2013). This task was adapted from Vogel, McCollough and Machizawa (2005). The task measures visuospatial working memory and is divided into three modes: two red rectangles, four red rectangles, and four red rectangles with two blue rectangles (distractors) shown against a black background screen. Participants are asked to remember the orientation of the red rectangles at two time points. The CDT score is calculated by a known formula (Pashler, 1988): K = S × (H – F)/(1 – F), where K is WMC, S is the set size of the array, H is the observed hit rate, and F is the false alarm rate. To use most of the data, we used the four rectangle K score as a representative CDT score for the Ducrocq et al. (2017) study.

**Far-Transfer Task:** Flanker Task (Berggren & Derakshan, 2013). This task is a modified version of Eriksen and Eriksen (1974) that is used to assess *inhibition*. Two types of arrows (distractor and target arrows) appear above or below the fixation mark, indicating right or left. Participants are required to focus on the target arrow and ignore the distractor arrows. During the first half of the trials, the arrows point in the same direction (congruent condition), while during the second half of the trials, the arrows point in opposite directions (incongruent condition). The flanker task also includes random bursts of white noise (noise condition) during the inter-trial interval in half of the blocks to manipulate anxiety state (Rossi & Pourtois, 2014). There was no noise in the other half block (safe condition). Interference scores were calculated by subtracting response times on compatible condition trials from times on incompatible condition trials. One of our datasets (Hotton et al., 2017) used a flanker task version with minor differences, such as X/N letters in place of the arrows. Moreover, this version of the flanker task did not include the noise condition. For the sake of using as much data as possible, we combined the interference scores for the safe and noise conditions across all our studies.

***Questionnaires***

**The Penn State Worry Questionnaire** (PSWQ; Meyer, Miller, Metzger, & Borkovec, 1990) is a 16-item self-report questionnaire developed to assess the trait of *worry*. The PSWQ demonstrated high internal consistency (α= 0.95) and test-retest reliability (α=0.92; Meyer et al., 1990).

**The Ruminative Responses Scale** (RRS; Nolen-Hoeksema & Morrow, 1991) is one of the subscales of the Response Styles Questionnaire that is used to measure the trait of *rumination*. The RRS consists of statements about self-focused responses to dysphoric mood, such as whether the participant feels sad or depressed. The RRS shows good internal consistency (Nolen-Hoeksema & Morrow, 1991).

**The State-Trait Anxiety Inventory** (STAI; Spielberger, Gorsuch, & Lushene, 1970) was designed to measure *anxiety* traits. The STAI consists of two subscale inventories: a state anxiety scale, indicating a more volatile state component, and a trait anxiety scale, indicating a more permanent trait. The STAI measures exhibited excellent internal consistency (α>0.89) (Barnes, Harp, & Jung, 2002), as well as convergent and discriminate validity with other measures (Spielberger, 1983).

**The Beck Depression Inventory-II** (BDI-II; Beck, Steer & Brown, 1996) is a well-known self-report inventory assessing the severity of depression. The BDI-II consists of 21 items ranging from 0-3. BDI-II has good convergent validity (r=0.71), high test–retest reliability (r =0.93) (Beck, Steer, & Brown, 1996) and high internal consistency (α=0.91) (Beck, Steer, Ball, & Ranieri, 1996).

**Sports Anxiety Scale-2** (SAS-2; Smith, Smoll, Cumming & Grossbard, 2006) is an instrument designed to measure sports anxiety. The scale is divided into three subscales, each inspecting different perspectives of sports anxiety: somatic, worry, and concentration disruption. The SAS-2 has been found to have adequate internal consistency (α=0.91) and fair test-retest reliability (r=0.87; Smith et al., 2006). Furthermore, convergent and discriminative validity tests indicated good validity for the questionnaire (Smith et al., 2006).

**Data Analysis**

Mental Health Indices

First, we standardized the raw scores of each of the questionnaires to Z scores. We used the statistical measures, i.e., means and standard deviations, that were reported in the earliest articles we found that examined validity and reliability of these questionnaires using large samples of the healthy population (STAI: Spielberger, 1983; BDI-II: Dozois, Dobson & Ahnberg, 1998; PSWQ: Meyer et al., 1990; RRS: Nolen-Hoeksema, Larson & Grayson, 1999; SAS-2: Smith et al., 2006). We used the corresponding statistical measures for female and male participants, when possible. As an exploratory step, we combined these two indices into one general mental state index to base the score on more data and examine a more general measurement of well-being.

Defining Learning Trajectories

The three main learning clusters are based on all available training trajectories, even the shorter ones. Only during the last training sessions in which some training trajectories are lack of training days due to shorter training duration, the training clusters are calculated based solely on the longer training trajectories.

**Table 1: Comparison of baseline, daily scores and slope** **between clusters**

In a comparison of the main training trajectories, a consistent difference in training score was found between training clusters. In addition, participants in the second and third training clusters exhibited a significantly greater improvement (slope) compared to the first training trajectory. Tukey tests compared all clusters to each other (i.e., cluster 1 vs. 2, cluster 2 vs. 3, cluster 1 vs. 3).

| **Day** | **Score** | | | **Anova (*Welch's Anova)** | **Tukey** | **Slope (based on data, not average)** | | | **Anova (*Welch's Anova)** | **Tukey** |
| --- | --- | --- | --- | --- | --- | --- | --- | --- | --- | --- |
|  | **Cluster 1** | **Cluster 2** | **Cluster 3** |  |  | **Cluster 1** | **Cluster 2** | **Cluster 3** |  |  |
| 1 | 1.39 | 1.86 | 2.46 | F(2,93)=43.73, p<0.001 | 2>1, p<0.001 3>2, p<0.001 |  |  |  |  |  |
| 2 | 1.33 | 2.24 | 2.95 | F(2,57.395)=94.471*, p<0.001 | 2>1, p<0.001 3>2, p<0.01 | -0.0334 | 0.3830 | 0.4900 | F(2,55.379)=18.6*, p<0.01 | 2>1, p<0.01 3>1, p<0.001 |
| 3 | 1.48 | 2.31 | 3.13 | F(2,57.959)=70.101*, p<0.001 | 2>1, p<0.01 3>2, p<0.01 | 0.1176 | 0.0657 | 0.1846 | F(2,56.637)=0.406*, p=n.s. | n.s. |
| 4 | 1.48 | 2.55 | 3.37 | F(2,93)=145.7 , p<0.001 | 2>1, p<0.001 3>2, p<0.001 | -0.0040 | 0.2576 | 0.2337 | F(2,92)=3.174, p<0.05 | n.s. |
| 5 | 1.5 | 2.56 | 3.51 | F(2,93)=184.4, p<0.001 | 2>1, p<0.001 3>2, p<0.001 | 0.0238 | 0.0122 | 0.1421 | F(2,93)=1.244, p=n.s. | n.s. |
| 6 | 1.62 | 2.74 | 3.57 | F(2,59.328)=154.16*, p<0.001 | 2>1, p<0.001 3>2, p<0.001 | 0.1225 | 0.1724 | 0.0661 | F(2,61.43)=0.663*, p=n.s. | n.s. |
| 7 | 1.61 | 2.94 | 3.64 | F(2,58.91)=196.32*, p<0.001 | 2>1, p<0.001 3>2, p<0.001 | -0.0090 | 0.2079 | 0.0669 | F(2,56.441)=1.994*, p=n.s. | n.s. |
| 8 | 1.76 | 2.95 | 3.71 | F(2,42.21)=104.17*, p<0.001 | 2>1, p<0.001 3>2, p<0.001 | 0.0755 | 0.0523 | 0.0722 | F(2,93)=0.033*, p=n.s. | n.s. |
| 9 | 1.68 | 3.06 | 3.69 | F(2,31.526)=90.28*, p<0.001 | 2>1, p<0.001 3>2, p<0.01 | -0.0656 | 0.1024 | -0.0189 | F(2,33.112)=0.813*, p=n.s. | n.s. |
| 10 | 1.69 | 3.12 | 3.78 | F(2,31.279)=128.66*, p<0.001 | 2>1, p<0.001 3>2, p<0.01 | 0.0033 | 0.0548 | 0.0839 | F(2,28.922)=0.164*, p=n.s. | n.s. |
| 11 | 1.53 | 3.21 | 3.78 | F(2,54)=157.1, p<0.001 | 2>1, p<0.001 3>2, p<0.05 | -0.0562 | 0.0689 | -0.0036 | F(2,25.049)=0.313*, p=n.s. | n.s. |
| 12 | 1.58 | 3.21 | 3.68 | F(2,54)=62.06, p<0.001 | 2>1, p<0.001 3>2, p<0.05 | 0.0436 | 0.0006 | -0.1001 | F(2,54)=0.368, p=n.s | n.s. |
| 13 | 1.44 | 3.3 | 3.76 | F(2,52)=115.8, p<0.001 | 2>1, p<0.001 3>2, p<0.05 | -0.1253 | 0.0971 | 0.0714 | F(2,28.544)=1.55*, p=n.s | n.s. |
| 14 | 1.43 | 3.2 | 3.8 | F(2,20.389)=120.83*, p<0.001 | 2>1, p<0.001 3>2, p<0.001 | -0.0016 | -0.0540 | 0.0621 | F(2,29.691)=0.650*, p=n.s | n.s. |
| 15 | 1.28 | 3.34 | 3.7 | F(2,24)=120.2, p<0.001 | 2>1, p<0.001 3>1  p<0.001 | -0.1383 | -0.0500 | -0.0321 | F(2,24)=0.198, p=n.s. | n.s. |

Reliability Test of Training Clusters


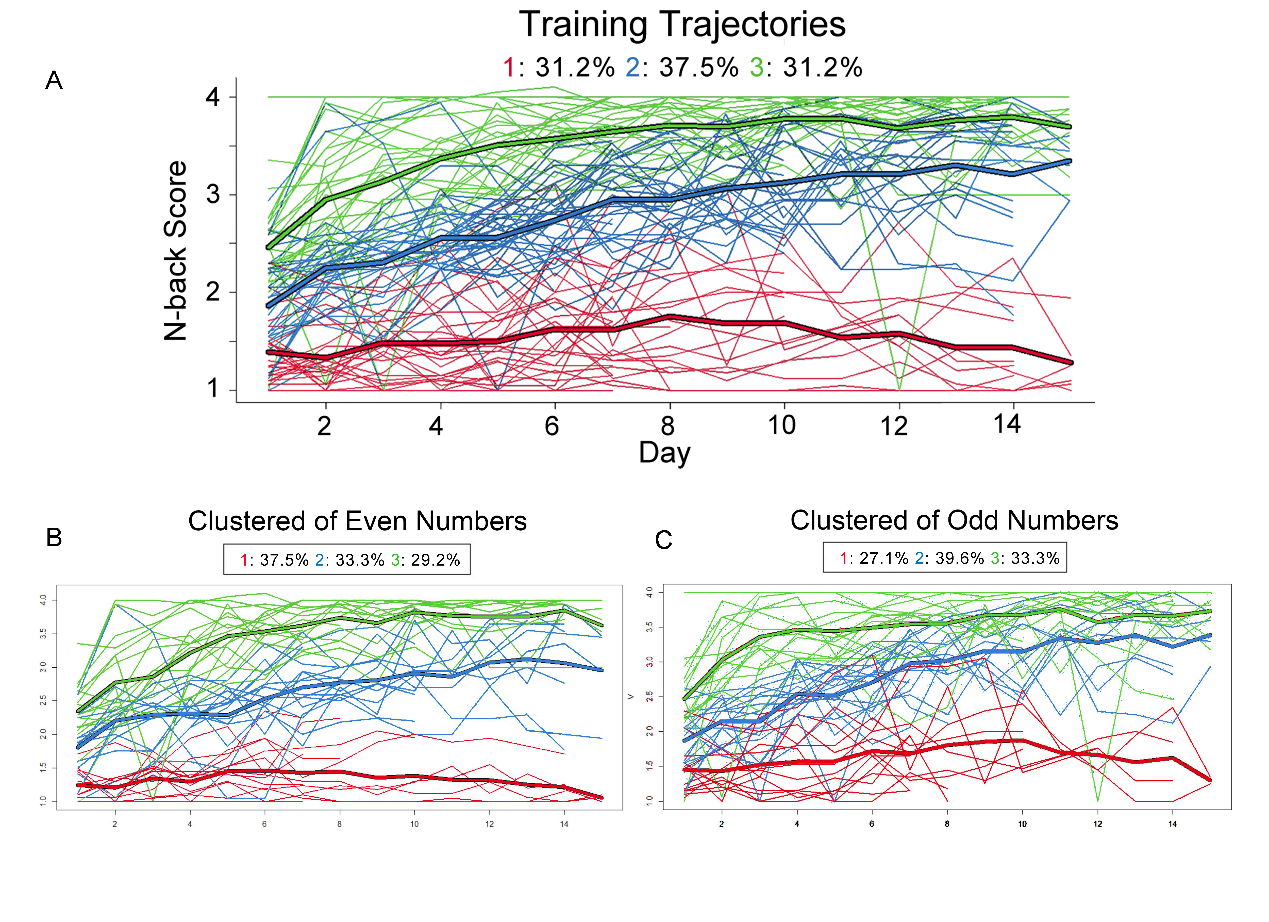


**Fig 1 Cross-validation reliability for Training Trajectories**

In both cross-validation training groups (B & C), the shape of all three clusters was similar to the clusters for the general training groups trained on all the data (A).

**Table 2: Using Study Number as Moderator for Training Outcomes**

|  | **Unstandardized Coefficients** |  | **Standardized Coefficients** | **t** | **Sig.** |
| --- | --- | --- | --- | --- | --- |
|  | **B** | **Std. Error** | **Beta** |  |  |
| **(Constant)** | **-0.301** | **0.164** |  | **-1.833** | **0.071** |
| **Is_study2** | **-0.003** | **0.183** | **-0.002** | **-0.018** | **0.986** |
| **Is_study3** | **0.022** | **0.185** | **0.018** | **0.119** | **0.906** |
| **Is_study4** | **-0.259** | **0.192** | **-0.179** | **-1.351** | **0.181** |
| **Is_study5** | **-0.273** | **0.174** | **-0.244** | **-1.573** | **0.120** |
| **Cluster** | **0.223** | **0.077** | **0.349** | **2.892** | **0.005** |

As the table shows, no specific study was found to predict training outcomes. Nevertheless, training cluster was significantly related to training results.

Mediation analysis

**Table 3: Mediation analysis results summary**

| **Regressions** |  |  |  |  |
| --- | --- | --- | --- | --- |
|  | Estimate | Std Error | Z value | P value |
| CDT Pre ~ Anxiety Improvement | 0.036 | 0.075 | 0.482 | 0.63 |
| Training Cluster ~ Anxiety Improvement | 0.157 | 0.083 | 1.893 | 0.058 |
| CDT Pre ~ Training Cluster | 0.283 | 0.096 | 2.94 | 0.003 |

| **Intercepts** |  |  |  |  |
| --- | --- | --- | --- | --- |
|  | Estimate | Std Error | Z value | P value |
| Anxiety Improvement | 0.329- | 0.179 | 1.838- | 0.066 |
| Training Cluster | 1.637 | 0.166 | 9.861 | 0 |

| **Variances** |  |  |  |  |
| --- | --- | --- | --- | --- |
|  | Estimate | Std Error | Z value | P value |
| Anxiety Improvement | 0.250 | 0.042 | 5.916 | 0.000 |
| Training Cluster | 0.559 | 0.087 | 6.403 | 0.000 |

Comparison of prognostic value of baseline and training trajectories to training outcomes

We found a correlation between the baseline score on the training trajectories (i.e., the score on the first training day) and improvement in anxiety symptom, R = 0.255, which can be converted to Cohen's d = 0.527. Similarly, the task score on the last training day was correlated with improvement in anxiety symptoms, R = 0.257, which can be converted to Cohen's d = 0.532. Yet, we also found that training trajectories had a bigger effect size on training outcomes with Cohen's f of 0.3388, which can be converted to Cohen's d =0.678.

Comparison of improvement in cognitive abilities between clusters

To examine whether clusters differed in improvement in each of the tasks, an ANOVA test was used. Both improvement in CDT score $[F_{\left( 2,65 \right)}=1.050, p=0.356. , \eta^{2}= 0.03]$ and flanker task $[F_{\left( 2,51 \right)}=2.370, p=0.104. , \eta^{2}= 0.09]$ did not differ between clusters. Paired-sample t-tests indicated that participants improved in CDT score $[t_{\left( 81 \right)}=4.779, p<0.001. , cohen^{'}s d=0.528]$ but not in flanker task $[t_{\left( 51 \right)}=0.350, p=0.972. , cohen^{'}s d= 0.005]$.

**References**

Barnes, L. L., Harp, D., & Jung, W. S. (2002). Reliability generalization of scores on the Spielberger state-trait anxiety inventory. *Educational and Psychological Measurement, 62*(4), 603-618. <https://doi.org/10.1177%2F0013164402062004005>

Beck, A. T., Steer, R. A., Ball, R., & Ranieri, W. F. (1996). Comparison of beck depression inventories-IA and-II in psychiatric outpatients. *Journal of Personality Assessment, 67*(3), 588-597. <https://doi.org/10.1207/s15327752jpa6703_13>

Beck, A. T., Steer, R. A., & Brown, G. K. (1996). Beck depression inventory-II. San Antonio, 78(2), 490-498.‏ <https://psycnet.apa.org/doi/10.1037/t00742-000>

Berggren, N., & Derakshan, N. (2013). Attentional control deficits in trait anxiety: Why you see them and why you don’t. *Biological Psychology*, 92(3), 440-446. <https://doi.org/10.1016/j.biopsycho.2012.03.007>

Dozois, D. J., Dobson, K. S., & Ahnberg, J. L. (1998). A psychometric evaluation of the Beck Depression Inventory–II. *Psychological assessment*, 10(2), 83. <https://psycnet.apa.org/doi/10.1037/1040-3590.10.2.83>

Ducrocq, E., Wilson, M., Smith, T. J., & Derakshan, N. (2017). Adaptive working memory training reduces the negative impact of anxiety on competitive motor performance. *Journal of Sport and Exercise Psychology*, 39(6), 412-422. <http://dx.doi.org/10.1123/jsep.2017-0217>

Eriksen, B. A., & Eriksen, C. W. (1974). Effects of noise letters upon the identification of a target letter in a nonsearch task. *Perception & psychophysics*, 16(1), 143-149. <https://doi.org/10.3758/BF03203267>

Hotton, M., Derakshan, N., & Fox, E. (2017). A randomised controlled trial investigating the benefits of adaptive working memory training for working memory capacity and attentional control in high worriers. *Behaviour research* and therapy, 100, 67-77. <https://doi.org/10.1016/j.brat.2017.10.011>

Jaeggi, S. M., Buschkuehl, M., Jonides, J., & Perrig, W. J. (2008). Improving fluid intelligence with training on working memory. *Proceedings of the National Academy of Sciences*, 105(19), 6829-6833. <https://doi.org/10.1073/pnas.0801268105>

Meyer, T. J., Miller, M. L., Metzger, R. L., & Borkovec, T. D. (1990). Development and validation of the Penn State worry questionnaire. *Behaviour research and therapy*, 28(6), 487-495. <https://doi.org/10.1016/0005-7967(90)90135-6>

Nolen-Hoeksema, S., & Morrow, J. (1991). A prospective study of depression and posttraumatic stress symptoms after a natural disaster: the 1989 Loma Prieta Earthquake. *Journal of personality and social psychology*, 61(1), 115.‏ [https://doi.org/10.1037//0022-3514.61.1.115](https://doi.org/10.1037/0022-3514.61.1.115)

Owens, M., Koster, E. H., & Derakshan, N. (2013). Improving attention control in dysphoria through cognitive training: Transfer effects on working memory capacity and filtering efficiency. *Psychophysiology*, 50(3), 297-307. <https://doi.org/10.1111/psyp.12010>

Pashler, H. (1988). Familiarity and visual change detection. *Perception & psychophysics*, 44(4), 369-378. <https://doi.org/10.3758/BF03210419>

Rossi, V., & Pourtois, G. (2014). Electrical neuroimaging reveals content-specific effects of threat in primary visual cortex and fronto-parietal attentional networks. *NeuroImage*, 98, 11-22. <https://doi.org/10.1016/j.neuroimage.2014.04.064>

Smith, R. E., Smoll, F. L., Cumming, S. P., & Grossbard, J. R. (2006). Measurement of multidimensional sport performance anxiety in children and adults: The Sport Anxiety Scale-2. *Journal of Sport and Exercise Psychology*, 28(4), 479-501.‏ <http://dx.doi.org/10.1123/jsep.28.4.479>

Spielberger, C. D. (1983). Manual for the State-Trait Anxiety Inventory STAI (" self-evaluation questionnaire").

Spielberger, C. D., Gorsuch, R. L., & Lushene, R. E. (1970). Stai. Manual for the State-Trait Anxiety Inventory (Self Evaluation Questionnaire). *Palo Alto California: Consulting Psychologist*, 22, 1-24.

Vogel, E. K., McCollough, A. W., & Machizawa, M. G. (2005). Neural measures reveal individual differences in controlling access to working memory. *Nature*, 438(7067), 500-503.‏
